# “Evil is Real and Attitude is Everything”: Applying Shattered Assumptions Theory to Worldview Changes Following Wrongful Conviction

**DOI:** 10.1002/bsl.70069

**Published:** 2026-05-29

**Authors:** Kathryn A. Thomas, Madeline R. Stenersen, Lily Hoerner, Chloe Kaminsky

**Affiliations:** 1 Department of Psychiatry & SEICHE Center for Health and Justice, Yale School of Medicine, New Haven, Connecticut, USA; 2 Department of Psychology, Saint Louis University, St. Louis, Missouri, USA; 3 Northeastern University, Boston, Massachusetts, USA; 4 University of Miami Miller School of Medicine, Miami, Florida, USA

**Keywords:** exonerees, posttraumatic cognitive changes, posttraumatic stress disorder, trauma, wrongful convictions

## Abstract

Wrongful convictions continue to occur at high rates. Research has revealed that negative posttraumatic cognitive changes are a risk factor for the development and maintenance of posttraumatic stress disorder, yet little research has examined whether exonerees experience posttraumatic cognitive changes, such as changes to their worldview. Thus, this study aims to understand exonerees’ self-reported life changes and worldview changes through the lens of the Shattered Assumptions Theory. Fifty-eight exonerees answered four open-ended questions about changes they experienced following their wrongful conviction, including changes to their worldview. Thematic analysis was used to identify themes. Exonerees reported a variety of positive (42.8%) and negative (78.5%) life changes, including changes to their worldview. The most commonly reported negative worldview changes included loss of faith in the legal system, people, and authority figures. Positive worldview changes included strengthened religious faith, learning that people are willing to help, and learning to control one’s attitude and focus on the positive. Results identified commonly reported worldview changes in exonerees, many of which are consistent with the Shattered Assumptions Theory. These findings from the largest qualitative sample of exonerees provides preliminary evidence for areas to be targeted in cognitive therapy in exonerees.

## 1 | Introduction

Wrongful convictions are a known and significant problem in the United States, with the number of exonerations increasing in recent years (Annual Report 2023). As of 2022, exonerees in the United States have collectively spent 2245 years incarcerated (Annual Report 2023). A wrongful conviction occurs when a person is convicted of a crime they did not commit, and an exoneration occurs “when a person who has been convicted of a crime is officially cleared based on new evidence of innocence” (Innocence Project 2025).

As the rate of exonerations has grown over the last 4 decades, research has explored the unique challenges that people who are wrongfully convicted face while incarcerated and during the reentry period (Annual Report 2023; Thomas and Hoyt 2023a, 2023b; Kukucka et al. 2022). Like other incarcerated people, exonerees report experiencing high levels of trauma exposure and related psychopathology while incarcerated (Baranyi et al. 2018; Kukucka et al. 2022; Thomas and Hoyt 2023a, 2023b). One study found the majority of exonerees in their sample witnessed (74.6%) and/or experienced (79.7%) one or more traumatic events while in prison, with the most common event being physical assault (Kukucka et al. 2022). Further, ample literature has also examined how incarceration can, in itself, serve as a traumatic event for those who experience it (Schnittker 2014).

While sparse and with small sample sizes, the existing literature suggests that exonerees endorse significant mental health concerns as a result of their wrongful conviction, including unique stressors beyond the stress of incarceration (A. T. Grounds 2005). People who are wrongfully convicted are often isolated in their claims of innocence, frequently experience multiple unsuccessful appeals, face longer sentences due to their refusal to admit culpability in the crime, and tend to have high profile or notorious cases (A. T. Grounds 2005). Further, many wrongfully convicted people become extremely focused on their legal appeals, resulting in detachment from their surroundings and social isolation within correctional institutions (Campbell and Denov 2006). It has also been suggested that people who are wrongfully convicted experience a unique trauma when trying to make meaning of their arrest and conviction when they know that they are innocent (A. T. Grounds 2005; Simon 1993; Thomas and Hoyt 2023a). This increased psychological burden comes from the known moral wrongfulness of their ordeal and the fact that their convictions were often caused by deliberate misconduct of police and/or prosecutors (Westervelt and Cook 2010).

Given the unique stressors experienced by exonerees, it is not surprising that exonerees face higher rates of mental health disorders than both the general population and prison populations in the United States (Wildeman et al. 2011). Although much of the existing research involves small sample sizes and occurs outside of the U.S., existing studies in the U.S. have found high rates of mental health disorders in exonerees, such that 27%–55% of exonerees met criteria for clinical depression, 39% for generalized anxiety, and 24%–66% for posttraumatic stress disorder (PTSD; Wildeman et al. 2011; A. Grounds 2004; A. T. Grounds 2005; Thomas and Hoyt 2023b). Although research has demonstrated high rates of mental health disorders in exonerees, little scholarship has explored how existing psychological theories can provide an understanding of why exonerees develop mental health disorders and how to effectively address the mental health needs in this vulnerable population.

One notable theory that can be used to understand how experiences of trauma can lead to worldview changes and related psychopathology is the Shattered Assumptions Theory (Janoff-Bulman 1992). According to the Shattered Assumptions Theory, trauma can shatter one’s assumptive world, which is the conceptual system that allows one to function effectively by providing expectations about oneself and the world (Janoff-Bulman 1992, 2004, 2010). Fundamental to this theory are three central worldviews encompassed within the assumptive world: (1) the self is competent, (2) people are benevolent, and (3) the world is just and fair (Janoff-Bulman 1992). When one’s assumptive world is broken down or shattered, they enter a hyper-aware state wherein they recognize their personal vulnerability and enter an unknown world where they are defenseless and, most often, terrified (Edmondson et al. 2011; Janoff-Bulman 1992). A shattering of the assumptive world can then lead to impaired functioning as a result of tainted expectations about the world, others, and self. Put simply, if someone does not have trust in people as benevolent, the world as good, and the self as competent, they are less likely to adapt to society in a psychologically healthy way. This theory is consistent with research examining the role of negative cognitive changes following trauma, such as negative changes to worldview, in the development and maintenance of PTSD (Dunmore et al. 2001; Ehlers and Clark 2000; Foa et al. 1999; LoSavio et al. 2017; Schuler and Boals 2016), related depression (Gonzalo et al. 2012), and suicidal ideation (Horwitz et al. 2018).

Posttraumatic cognitive changes are a key therapeutic target in a variety of psychological interventions, including Cognitive Processing Therapy (CPT) and trauma-informed CBT (TF-CBT; American Psychological Association 2025). CPT and TF-CBT are gold standard treatments for PTSD and recommended by the American Psychological Association (APA) as well as the United States Department of Veterans Affairs (American Psychological Association 2025). While CPT is not based on Shattered Assumptions Theory, the two are intricately connected. CPT involves changing both the content of cognitions and the impact cognitions have on behavior. According to CPT, when people try to integrate their traumatic experiences into existing cognitive structures or ways of thinking, they can do it one of three ways. The first way is through *accommodation*, which is the ultimate goal of CPT; this involves integrating accurate information about the memory of the experience with beliefs about the assumptive world (i.e., “I made the best decisions I could with what I knew then. I was wrongfully convicted due to the actions of prosecutors, not because I deserved it.”). The other maladaptive ways of combining memories from traumatic events into existing ways of thinking are *assimilation* and *over-accommodation. Assimilation* involves integrating the traumatic experience to match prior beliefs about the world (e.g., “If I had remembered every detail of what happened, the police wouldn’t have believed I was lying.”), while *over-accommodation* is altering one’s beliefs about the world in an extreme way to feel a sense of control in the future (e.g., “I experienced police misconduct and now I can never trust law enforcement.”). All forms of cognitive behavioral therapy, including CPT, tend to involve labeling and reframing thoughts or cognitions as a way of addressing psychological distress. Overall, there is significant overlap between the Shattered Assumptions Theory and CPT, as both describe how traumatic experiences impact one’s world beliefs, and ways that changes in world beliefs may contribute to psychological distress. In order to provide effective mental health treatment for exonerees, it is critical that researchers and clinicians consider how existing theories and treatments can be applied, and adapted as needed, to meet the unique needs of this population. In connecting Shattered Assumptions Theory to core components of validated clinical treatments for PTSD, our aim here is to gather the posttraumatic worldviews of this unique population to inform treatment approaches that target cognitive worldviews.

Despite the importance of understanding negative posttraumatic cognitive changes as a potential risk factor for the development and maintenance of mental health disorders, little research has examined how being wrongfully convicted can change one’s worldview (Thomas and Hoyt 2023a, 2023b). Although the Shattered Assumptions Theory has not yet been applied to wrongful convictions, researchers have posited that the theory has implications for how individuals perceive interactions with societal institutions such as police (Posey et al. 2026). Thus, we posit that the Shattered Assumptions Theory will provide a helpful framework to understand how exonerees experience changes in beliefs about themselves, other people, and the world more generally.

Thus, this study aims to understand exonerees’ self-reported changes in their worldview as a result of their wrongful conviction through the lens of the Shattered Assumptions Theory. Ultimately, we aim to gain a preliminary understanding of worldview changes in exonerees, with the goal of adapting evidence-based mental health treatments to meet the unique needs of exonerees and inform cognitive targets of existing PTSD treatments for this population. In addition to asking directly about worldview changes, we also asked participants to describe their life changes as a result of their wrongful conviction more generally, as understanding and addressing psychosocial needs is an essential part of effective mental health treatment.

## 2 | Methods

### 2.1 | Participants

A total of 74 participants were recruited through a listserv of people who were wrongfully convicted and subsequently exonerated, to complete an online survey on Qualtrics, a secure online survey platform. In addition to a variety of validated scales, the survey contained four optional open-ended qualitative questions, which are the focus of the current study. A total of 58 participants completed at least one of the open-ended qualitative questions. The overall goal of the survey was to assess self-reported mental health symptoms in exonerees, as well as factors associated with positive and negative mental health outcomes. The listserv is a Google Group that exonerees can elect to join when they attend the annual Innocence Network Conference. The survey was reviewed and determined to be exempt by the University of Wisconsin-Madison Institutional Review Board. Additionally, the Research Committee at the Innocence Network reviewed and approved the survey. Members of the Exonerees Google Group were invited to participate in the study via an email. Participants were provided a $20 gift card to Amazon to thank them for their participation.

Of the 58 participants who completed the qualitative questionnaire, the majority of participants were male (*n* = 44, 75.9%), White (*n* = 38, 65.5%), in a relationship or married (*n* = 40, 69.0%), currently employed (*n* = 30, 51.7%), and identified as religious/spiritual (*n* = 40, 69.0%). See Table 1 for the demographic characteristics of participants. In terms of their experience with the legal system, the majority of participants were convicted in their first trial (*n* = 45, 77.6%), had a private attorney (*n* = 34, 58.6%), were not released on bond (*n* = 24, 41.4%), had five or more appeals prior to exoneration (*n* = 27, 46.6%), and were exonerated in state court (*n* = 50, 86.2%). See Table 2 for a summary of legal variables. Participants spent an average of 15.2 years in prison prior to their exoneration, with a range of 2–37 years (SD = 8.24).

### 2.2 | Procedure

The comprehensive procedure and results from other components of this study on exonerees can be found elsewhere (Thomas and Hoyt 2023a, 2023b). As part of an online Qualtrics survey, participants completed a series of four open-ended questions, adapted from Kaler (2009). The goal of the qualitative questions was to gain a deeper understanding of exonerees’ experiences of posttraumatic stress, posttraumatic growth, and worldview changes. The questions included:

1. How did your life change as a result of your wrongful conviction?
2. Sometimes people say that bad events have led to a variety of problems in their lives. Was this true for you? What types of problems did you observe?
3. Sometimes people say that bad events have led to positive changes in their lives. Was this true for you? What types of positive changes did you experience?
4. Sometimes people say that bad events resulted in changes to their most basic beliefs about the world, the things they assumed to be true about the world. Was this true for you? What changes to your most basic beliefs did you experience?

We chose to collect qualitative data in an open-ended survey format. Researchers have posited that open-ended survey questions may mitigate the effects of social desirability bias and allow participants to share information more openly, especially when discussing sensitive or personal topics (Sheldon 2017; Albudaiwi 2017; Fisher 1993).

### 2.3 | Data Analysis

Thematic analysis was used to extract themes of exonerees’ experiences. Specifically, coders identified patterns of meaning through an inductive approach within the written responses in accordance with thematic analysis guidelines discussed by Braun and Clarke (2012). First, all written responses to the open-ended question were reviewed and coded for meaning by two coders independently (first and second author). The two coders then met and came to consensus on major themes and subthemes of the data from the coded transcripts. Of note, though data represents responses to several written-response questions, participants’ responses to these different questions often contained codes across multiple themes. This means results map onto the themes identified in the coding process rather than discrete answers to each of the questions. In the event of a disagreement between two coders, a third coder was included to discuss the item until an agreement was reached. The coding team consisted of two early career faculty who graduated from counseling psychology PhD programs and one master’s student in a community psychology program.

## 3 | Results

A complete list of themes that emerged for each of the four qualitative questions are summarized in Table 3. Themes will be summarized and discussed for each question.

### 3.1 | Question 1. Life Changes as a Result of Wrongful Conviction

First, participants were asked, “How did your life change as a result of your wrongful conviction?” Participants’ responses were coded, and themes were categorized as positive or negative changes. Of the 56 participants who completed Question 1, 27 (48.2%) reported only negative changes, 7 (12.5%) reported only positive changes, 17 (30.3%) reported both positive and negative changes, and 5 (8.9%) reported only neutral changes.

#### 3.1.1 | Negative Changes

Participants reported a variety of negative changes as a result of their wrongful conviction. In total there were 10 themes of negative changes that emerged including (1) Loss of Relationships; (2) Loss of Parenting; (3) Structural Barriers; (4) Financial Problems; (5) Negative Emotions and Psychological Changes; (6) Grief and Loss; (7) Negative Changes to Worldview; (8) Disgraced Reputation; (9) Health Problems; and (10) Arrested Development/Loss of Formative Years. The number of participants who noted responses within each theme is reported in Table 3.

The most common negative theme that emerged across participants was Loss of Relationships and overall social challenges after their exoneration. A total of 27 participants (48.2%) described a variety of challenges in relationships, including feeling less close to others, difficulty trusting others, loss of family, experiencing the death of loved ones while incarcerated, the end of romantic relationships and friendships, and estranged relationships with their children. Several participants described their challenges in relationships as the most negative impact of their wrongful conviction. For example, one participant stated, “My most negative impact is my inability to love people, or enter into relationships on any level.” Another participant described how their wrongful conviction led to challenges trusting other people:

I do not socialize anymore, I don’t do a fraction of the things I used to enjoy doing. I feel people are talking about me behind my back, and that I am being lied to by nearly everyone I know and used to trust. Everyone seems to be unstable, and unpredictable to me. […] The only time I feel normal and OK, is when I’m home with the curtains and doors closed. Away from people. I love people, though. I’d help anyone in any way I can, and wish no evil upon anyone. I just feel I’ve been lied about, taken advantage of, stolen from and treated like dirt my entire life. People have not proven to be a great thing in my life. But I do have friends, but can’t seem to get close to anyone anymore due to not really trusting people anymore. That’s the big change in my life: I have a very hard time trusting anyone now. When I let my guard down, I feel like I’ve made a big mistake. It’s a mess with trust.

Another type of loss described by participants was Loss of Parenting. A total of 12 participants (21.4%) described the negative impact of their wrongful conviction on their ability to parent their children. For example, one female participant stated, “I was not able to raise my children and I missed a lot of important milestones in their lives.” Another female exoneree described how she lost the ability to have children as a result of her wrongful conviction: “My dream of having another child was squashed because my age made it impossible and I could not afford medical advancements to help.” One participant described how her wrongful conviction led to her “kids gr[o]w [ing] up in foster care with abusive foster parents.”

Another common theme that emerged was Structural Barriers faced when re-entering society upon release, including challenges with housing, barriers to employment, loss of career, financial problems, and a general lack of resources. A total of 18 participants (32.1%) described experiencing structural barriers upon re-entry following exoneration. Several exonerees (*n* = 5; 8.9%) described experiencing Financial Problems as a result of their wrongful conviction. Many exonerees experienced financial stress upon re-entry, with one participant stating, “Before I never worried about finances, now it is a constant struggle to get by.” The legal costs incurred throughout their wrongful conviction led to lasting financial consequences for many exonerees. One participant shared, “financially, things are a lot more harder now due to the costs expended in the case.” Despite being exonerated, many participants discussed how their history of incarceration led to challenges obtaining employment: “Since release, I have been turned down for jobs that I feel very qualified for, turned down due to having been incarcerated. Once people know I have been in prison, they seem to step back a little.” Many participants lost careers that they had worked their entire lives to build. Another participant described the profound lack of resources and support she experienced upon re-entry:

I realize that I have been hardened by the wrongful conviction and that hasn’t lessened after my release. The things that I wanted when I left prison were out of my reach. There was no money for continuing my education. I had to focus on getting a home, rebuilding my life and caring for my 16 year old son that I gave birth to in prison. I couldn’t get married because I no longer trust people. I barely date. No one wants to stick around and hope that I get over my issues. […] Literally, I walked out of prison with nothing but my prison uniform. I had to rebuild everything from scratch and my dreams/hopes were dismissed.

A total of 10 participants (17.9%) described experiencing Negative Emotions and Psychological Changes as a result of their wrongful conviction. For example, one participant described the mental health impacts he experienced upon release as follows:

Well I was not a prisoner anymore—I could do what everyone who was free was doing. Emotionally, I was a wreck—very happy to have been freed but left to this world with nothing after 27 years. If not for people helping me, I would not have had anything upon my release.

Another theme described by exonerees related to their mental health was a profound sense of Grief and Loss, including loss of time, opportunities, and experiences. A total of 15 participants (26.7%) described a sense of Grief and Loss. One participant described this as “a sense of loss that doesn’t go away.” This participant described the ongoing impact of losing time due to their wrongful conviction:

When I was arrested, I was 17 years old. When I was released on parole, I was 42 years old. I have a sense of loss that doesn’t go away. I was a high school senior with my youth ahead of me. Now I’m a middle aged man with my youth behind me.

Several exonerees (*n* = 5; 8.9%) described how their wrongful conviction led to Negative Changes to their Worldview, including decreased faith in humanity and the legal system. One exoneree described how his wrongful conviction changed his perception of the legal system as follows:

I have completely lost faith in the legal system. I learned that “experts” aren’t experts at all, that the prosecution is only out to get a conviction, and that the police and prosecutors are nothing but a brotherhood that will do everything they can to protect each other

Several exonerees described a loss of faith in humanity more generally. For example, one exoneree described: “I lost faith in a higher being, law enforcement, the belief in people as being good. Conversely, I learned I could change for the better and break the cycle of dysfunction within one’s family and thus, within society.”

A total of 5 participants (8.9%) experienced a Disgraced Reputation as a result of their wrongful conviction. Exonerees described how the high profile nature of their case led to ongoing harms to their reputation. One participant stated, “I went from being a private person to a public person. Nothing I will ever accomplish in life will come to define me more than my proximity to a horrific murder that I didn’t commit.” Another participant described the impact of the media on their identity: “My case was in the international news for nearly a decade. I continue to be vilified in the tabloids. My every effort to reclaim my identity and my life is a battle.” Another participant succinctly stated, “My reputation is tarnished.”

A total of 5 participants (8.9%) described how their wrongful conviction had negative impacts on their health. One participant described the impact of stress on his health: “I think that all the stress and worry of these events have made me sick in many ways health wise.”

A less common but notable theme was missing formative years and a sense of arrested development. One participant described a sense of arrested development, such that he felt stuck at the age in which he was incarcerated rather than his actual age upon release. Specifically, one participant stated, “I feel like a man out of time. I missed so much. I still feel like I’m 24 in my head, like I missed formative years that help one develop and grow as a person socially and emotionally.” Two participants described how their exoneration led to loss of their “formative years.”

#### 3.1.2 | Positive Changes

Participants also reported positive changes as a result of their wrongful conviction. Themes of the positive changes noted by participants included (1) Positive Psychological Changes; (2) Increased Appreciation of Life; (3) Improved Relationships; (4) Positive Emotions; (5) Greater Sense of Meaning; (6) Increased Spirituality; (7) Positive Career Changes; and (8) Became a Better Person.

The most common positive theme reported by participants was Positive Psychological Changes (*n* = 11; 19.6%), which included Increased Appreciation of Life (*n* = 8; 14.2%), Positive Emotions (*n* = 3; 5.4%); and Greater Sense of Meaning (*n* = 2; 3.6%). One participant described his increased appreciation for life as follows:

The biggest change in my life is the depth of my appreciation of everything. Life certainly gets hectic at times, but because of the experience I am able to throttle down the pace and respond appropriately.

Another participant described how his wrongful conviction taught him to cherish his life upon release: “Considering that I was supposed to die in prison, it’s like what the devil meant for evil, God turned it around for my good. Prison taught [me] to cherish every moment of my life and never take things for granted.”

Although one participant described a loss of spirituality as a result of his wrongful conviction, other participants (*n* = 8; 14.2%) described how their wrongful conviction and subsequent exoneration led to Increased Spirituality. One participant described how their wrongful conviction “forced me into a very deep and depending relationship with God and the Spiritual World.” Another participant stated, “I changed my life from drugs and alcohol to faith in God.”

While many participants described challenges with relationships upon release, other participants described Improved Relationships (*n* = 7; 12.5%), including increased closeness with others (*n* = 3; 5.4%) and an increased appreciation of family (*n* = 4; 7.1%). For example, one participant stated, “I became even closer with family and friends that supported me.”

Several participants (*n* = 5; 8.9%) described how their wrongful conviction led to Positive Career Changes, including several exonerees who began careers with a goal of criminal legal reform:

It allowed me to speak from a position of authority about Criminal Justice issues to students, Lawyers and Investigators around the country. I wanted to share the truth about what was done wrong to me and no one can say I’m wrong about anything.

Two participants (3.6%) believe that they Became a Better Person as a result of their wrongful conviction: “Overall I think I am a better person capable of empathy that I wasn’t before.” Another participant described how he made himself a better person as a result of his wrongful conviction:

It would be an exercise in futility to even try to concisely, clearly, sufficiently! convey the multitude and magnitude of the changes as a result of my wrongful arrest/prosecution/conviction/incarceration and post imprisonment adjustment in writing alone. However. In simplistic overall terms I can tell you this: It made me a better person. Or should I say, I made me a better person? IN SPITE of what’s designed to crush…

#### 3.1.3 | Positive and Negative Changes

It is also important to note that some participants (*n* = 17; 30.3%) described a variety of both positive and negative changes as a result of their wrongful conviction. For example, one participant described a variety of life changes he experienced as a result of surviving his wrongful conviction and incarceration:

Despite dashed college plans at 22 upon my arrest, I survived 18 years, meeting my wife and having children & grandkids together. I educated myself every year, using this to earn my bachelor’s (summa) upon my exoneration. This helped me lecture globally, strengthen my commitment to family responsibilities while working to improve our world by also operating my commercial real estate investments and finishing my memoir.

#### 3.1.4 | Neutral Changes

Finally, several participants described changes that were neutral in nature, specifically changes that happened on “Every Level” of life. One participant described this change as follows: “Every opportunity and relationship in my life is impacted by my wrongful conviction.”

### 3.2 | Question 2. Problems as a Result of Wrongful Conviction

Participants were asked the second question of, “Sometimes people say that bad events have led to a variety of problems in their lives. Was this true for you? What types of problems did you observe?” Five themes resulted from the responses to this question including (1) Mental Health Consequences; (2) Structural Barriers; (3) Social Problems; (4) Stigma; and (5) None.

#### 3.2.1 | Mental Health Consequences

A total of 31 participants (55.4%) reported varying mental health consequences as a result of their wrongful conviction and incarceration. These included both internal feelings as well as externalizing behaviors related to their mental health. Internal mental health feelings and consequences noted included loneliness, numbness, pain, PTSD, sleep problems, loss of happiness, anxiety, depression, loss/grief, lack of life satisfaction, lack of patience, isolation, problems with confidence, and overall increase in stress. One participant noted how changes in their mental health due to their wrongful conviction impacts how they see others:

Anxiety and depression. Fearfulness of people. Nervousness. Always questioning people’s motives even when they are legitimate.

Externalizing behavioral consequences mentioned included substance use, anger issues, problems with focusing, and “workahol[ism].”

#### 3.2.2 | Social Problems

Social problems following incarceration were mentioned by a total of 22 participants (39.3%) and focused on problems related to internal social engagement and identity as well as external social relationships and interactions with others. Subthemes include Trust Issues (*n* = 5; 8.6%) and Estrangement (*n* = 2; 3.4%). One participant noted the consequences to his family and marriage: “Family conflict and dysfunction continues as [a] wedge between my wife and others envious of us.” Another noted a more universal loss of trust and faith: “Less faith in people and no trust in government.” Finally, one participant noted an overall disgust with some people and authority figures: “There are some very, very bad and disgusting people in the world, some of whom are in public office—which is what the bad experience of a wrongful conviction has taught me.”

#### 3.2.3 | Structural Barriers

Structural barriers noted by participants (*n* = 20; 35.7%) centered around barriers to re-establishing their lives and identity after their exoneration. Specific structural barriers mentioned (i.e., subthemes) include Financial Problems (*n* = 10; 17.2%), Barriers to Housing (*n* = 4; 6.9%), Barriers to Healthcare (=1; 1.7%), Barriers to Employment (*n* = 4; 6.9%), Loss of Work (*n* = 4; 6.9%), and Unhelpful Therapy (*n* = 2; 3.4%). One of the most frequent themes, Barriers to Employment, was summarized by one participant in the following way:

Been home three years on parole and dealing with curfews and constant monitoring. This makes me feel like I’m still a prisoner to a false conviction. I have been home three years and have only acquired temporary jobs even though I earned a Bachelor’s degree in Mathematics when I was in prison.

Another participant noted the intersecting barriers of healthcare and employment during re-entry:

I did not have a life[.] [W]hen I was released, it was very hard to get employment, very hard to find a place to live, very hard to get basic necessity care such as medical and dental and vision. [W]hen you are a non person for a quarter of a century it is very hard to re enter society.

#### 3.2.4 | Stigma

Stigma was noted by six participants (10.7%) as a negative consequence of their wrongful convictions. Stigma encompassed experiences of stigma against them from both broader society and those in their interpersonal network. In their summarization of the negative consequences of their wrongful conviction, one participant focused not only on the stigma experienced, but also how they rose above that stigma:

I certainly had to make adjustments to the stigma of being a convicted felon, but I took it in stride being confident that my own merits would rise above it.

Another participant noted stigma related to the disgraced reputation they experienced as a result of their wrongful conviction and the subsequent consequences of this:

I have suffered immense damage to my reputation and a great degree of loss of privacy. I feel anxiety and depression like I never did before. I feel fear and anger like I never did before.

#### 3.2.5 | None

Finally, eight participants (14.3%) noted “None” when asked about the negative consequences of their wrongful conviction/incarceration.

### 3.3 | Question 3. Positive Life Changes as a Result of Wrongful Conviction

For the third question, participants were asked, “Sometimes people say that bad events have led to positive changes in their lives. Was this true for you? What types of positive changes did you experience?” Eight themes resulted from the responses to this question including (1) Mental Growth; (2) Relational Growth; (3) Purpose; (4) New Skills; (5) Spiritual Growth; and (6) None.

#### 3.3.1 | Increased Mental Growth

A total of 16 participants (28.6%) expressed increased feelings of mental growth, including experiences of mental strength, inner peace, improved outlook, gratitude, resilience, confidence, and patience. In terms of increased patience, one participant reported, “Death row taught me to be patient. Also taught me that everything can be snatched away in a heartbeat and not a damn thing you can do.” Another participant described how, as a result of his wrongful conviction, “I’m more patient and understanding.” One exoneree described his feelings of gratitude following his exoneration as follows: “The appreciation of life is so great now. I hate that I lost 11 years, but I am most grateful for where I am now.”

#### 3.3.2 | Relational Growth

In connection with their wrongful conviction, 14 participants (25.0%) endorsed Relational Growth, which included gaining appreciation for loved ones, becoming a more understanding and loving person, gaining support from loved ones, developing a deeper compassion for others, developing closer family relationships, having more empathy for others, and overall more positive relationships with others. One participant noted her relational growth in this way: “I learned how to recognize who I can trust and the value of fewer, deeper, intimate relationships.”

One participant described how she “gained a lot more caring people” in her life since being exonerated. Other participants described their increased appreciation for family, stating “I learned the importance and value of a good family and friends” and “I appreciate family and justice.” Another exoneree remarked on how his wrongful conviction led to changes in his view of humanity:

I learned a lot about the legal system, prisons, people in prisons (staff and inmates), the folly of the courts and incompetence of the average attorney … but mostly, that there are still good people out there who, though you don’t even know them, will dedicate a part of their life to helping correct an injustice. There are still good people in the world, is what the bad experience of wrongful conviction has taught me.

Three participants (5.4%) expressed feelings of greater empathy for others and awareness of societal issues. One participant exemplified this theme by stating:

I came to understand my own resilience and perspective. Despite my feelings of alienation, I feel a deep compassion for anyone who suffers—innocent and guilty alike. I feel purposeful sharing that perspective and showing compassion to those others vilify.

Another participant described how he gained respect for others and learned to care for others who cannot care for themselves:

Gained a sense of caring for those who cannot care for themselves. Gained a respect for people feeling that all deserve respect regardless of their race or gender. Made me a more loving person because I went through so much pain and suffering.

#### 3.3.3 | Purpose

Twelve participants (21.4%) described an increased sense of Purpose in Life. Specifically, exonerees described experiences of increased overall meaning and purpose, purpose in work, and newfound perspective. One participant described how he “found purpose in life” and “inner peace.” Another participant described how, as a result of her wrongful conviction, she “developed purposefulness in my work.”

#### 3.3.4 | New Skills

Twelve participants (21.4%) noted that their wrongful conviction has led to them gaining New Skills, both in their career and personal life, including new educational opportunities, improved career opportunities, improved leadership skills, and new pathways in their life. Several exonerees reported that they became educated in prison, including one participant who stated, “I became educated in prison. I learned to think outside the paradigm I was raised in. I learned to be introspective and to think for myself.” Several participants described how their wrongful conviction led to new career paths related to criminal legal reform. One participant stated that he “studied law and became a lawyer, greatly broadening the horizons of my knowledge and life experience.” Another participant described a similar path to becoming an attorney following his exoneration: “I became an attorney and have met people that will help me make a difference to the state and potentially the country. I did not have the resources or network before my conviction that I have now.”

#### 3.3.5 | Spiritual Growth

Eleven participants (19.6%) described a stronger sense of spirituality. Several exonerees used the phrase “spiritual growth” to describe how their wrongful conviction led to changes in their spirituality. One participant described how his wrongful conviction led to a “closer relationship with my GOD.”

#### 3.3.6 | None

Ten participants (17.8%) noted no positive changes as a result of their wrongful conviction.

### 3.4 | Question 4. Worldview Changes as a Result of Wrongful Conviction

Below we discuss the themes seen in participants’ responses to the following question: “Sometimes people say that bad events resulted in changes to their most basic beliefs about the world, the things they assumed to be true about the world. Was this true for you? What changes to your most basic beliefs did you experience?” Overarching categories of the data that emerged include the mention of changes to worldview that were either negative or positive. Themes related to negative changes to worldview are presented first in order of frequency, followed by positive changes to worldview in the same order. A complete list of themes that emerged are summarized in Table 3.

#### 3.4.1 | Negative Changes to Worldview

Themes regarding negative changes to participants’ worldview broadly centered on either a loss of some kind (e.g., faith, trust), or something negative participants learned about the world they had not known or considered before. Themes included in this category are (1) Loss of Faith in Legal System, (2) Loss of Faith/Trust in People, (3) Loss of Faith/Trust in Authority Figures, (4) Loss of Religious Faith/Challenges to Religious Beliefs, (5) Learned about Wrongful Convictions/Problems with the Legal System, (6) Learned that People are Motivated by Self Interest, and (7) Learned that Life is Not Fair and the World is Not Safe.

Loss of Faith in the Legal System: A total of 11 participants (21.2%) described a loss of faith and trust in the legal system including courts, attorneys, law enforcement, and the legal system as a whole. One participant described how his mistrust of the police and legal system impacts other areas of his life:

I think I am more distant from the world. I do not interact with others. I think the major issue is trust in the police and judicial system. It was like walking down some stairs and suddenly one is missing. You begin to fall and you don’t land until many years after the prison sentence ends. When you do finally land, you are in an entirely different place. I believe after 24 years I am still adjusting. Maybe that is just the way life can be for some people.

Other participants noted specifically losing trust in the legal system as a result of their wrongful conviction, describing the system as “cold,” “evil,” and one that “destroys people, families, businesses, on a daily basis as part of routine.” One participant noted this decrease in trust in the legal system, despite having previously worked a police officer:

I am less trusting of people and the criminal justice system. Even though I was a police officer prior to my wrongful conviction, I did not believe an innocent person could be wrongfully convicted. I rely on God and my family.

Loss of Faith/Trust in People: A total of eight participants (15.4%) reported a loss of faith or trust in people, meaning that the participants’ view of humanity changed in a negative way as a result of their wrongful conviction. These responses often coincided with loss of faith or trust in the legal system, as participants described losing faith in humanity after realizing the harms committed by individuals within the legal system. One participant summarized his loss of faith in people as follows:

I learned that one does not have to love their parents just because they brought them into this world. I experienced that people are selfish, egotistical, self-promoters, and that most live fake lives.

Loss of Faith/Trust in Authority Figures: In addition to a general loss of trust in people as a whole, 7 participants (13.5%) noted a specific loss of trust in those in positions of authority. Authority figures mentioned included judges, politicians, media, legislators, and clergy among others. This loss of trust was often mentioned in the relation to the truth, as several participants described a belief that authority figures do not care about seeking that truth:

I know that authorities in crimes don’t seek the truth always, but blame is what they seek, and even when facts are presented, they are still in denial to the truth.

Similarly, others mentioned the power of authority figures to inflict pain and suffering on those who have less power:

I became more aware that the agenda of people in authority is inflicted on those who can neither resist or escape.

Loss of Religious Faith/Challenges to Religious Beliefs: Religious faith and belief systems were consistently mentioned by participants when reflecting on what had changed for the worse as a result of their wrongful conviction. Several participants mentioned a complete abandonment of religion and their faith:

I abandoned religion when I felt a just God would not allow an innocent man to go to prison.

Other participants noted how this change in religious faith also expanded to include a newfound lack of purpose in life itself:

I wasn’t sure how I felt about the existence of God going into this experience. Now I know God doesn’t exist. I now understand that there is nothing inherently moral in nature, and that the only purpose I can have in my life is what purpose I find for myself.

Another participant wrote:

I was agnostic before my wrongful conviction. I am now atheist. I understand that the universe has no fundamental purpose or meaning, and that human beings aren’t special.

Finally, others mentioned how, when put to the test during their wrongful incarceration, their religious beliefs and experiences did not hold up and their faith lessened:

Yes, I had strong Christian beliefs predicated on what I was taught. Essentially, 90 percent of what I believed, I no longer believe. I came to realize that so much of the spiritual or religious concepts and beliefs were based on what was convenient for the group. But once those beliefs were challenged by true adversity, it fell apart. I only was left with those beliefs that transcend, people, social class and geographical location. My statement became, if it doesn’t work for someone in a third world country as well as for those that are more fortunate here, then it really wasn’t worth investing in.

Learned about Wrongful Convictions and Problems with the Legal System. Participants mentioned several lessons they learned as a result of their wrongful convictions which fit within several themes. The first centers around what participants learned about wrongful convictions and the legal system while being forced to navigate it through their wrongful conviction and incarceration:

I never thought about wrongful convictions, or knew of the size of the problem like I do now. I learned more about governments, and how the people are treated.

Learned that People are Motivated by Self-Interest: Another commonly-reported lesson learned by participants was that people are motivated by their own self interest, only look out for themselves, and will hurt others in pursuit of those interests. One participant described how he previously had faith in a higher power, but now believes that there is no higher power, only people who are motivated by self-interest:

Faith in a higher power. There is no higher power. There are people in power and they are mostly only looking out for themselves.

Another participant noted explicitly that people are concerned with their own self-interest but went on to explain how he is choosing to live his life for others regardless of this belief:

Most people are only concerned with their own self-interest. However, I also chose not to live in this manner. I chose to live a life for others. I’ve made many sacrifices so that my children and others will not have to experience what I have lived through. I have chosen to change myself for the better and hopefully, foster positive change in others through my example.

Learned that Life is Not Fair and the World is Not Safe: The final theme in this category focuses on negative changes in worldview related to the world itself. Participants specifically noted learning through the process of their wrongful conviction that the world is not safe and that life is not fair. One participant described how he navigates the world since his wrongful conviction:

Evil is real and attitude is everything. This hardens me to navigate this world.

Another participant described the impact of their wrongful conviction on their worldview:

The changes in my basic beliefs about the world all came to me as a result of being wrongfully convicted.

##### 3.4.1.1 | Positive Changes to Worldview.

Though less frequent than negative changes, themes regarding positive changes to worldview centered around religious faith, people, and attitudes. Themes noted in this category include (1) Strengthened Religious Faith, (2) Learned that People are Willing to Help, and (3) Learned to Control Attitude/Focus on Positive.

Strengthened Religious Faith: Although some participants described negative impacts to their spirituality, several other participants noted positive changes related to their faith, with some citing that their faith was strengthened as a result of their wrongful conviction: “This bad event allowed me to strengthen my faith and belief in God.” Another participant wrote, “I became spiritual and learned to trust God.”

Learned That People Are Willing to Help: Several participants learned that people are willing to help during difficult times. This theme represents responses that take a positive view of people as a whole, highlighting that all people are not bad and remembering the moments in which participants were helped by others:

That all people in positions of power are not bad people. There are those who are willing to assist you.

Learned to Control Attitude/Focus on Positive: The final theme represents participants who learned to control their response to difficult situations by shifting their focus to the positive. Multiple participants mentioned a sense of control over how they live their lives since their exoneration and how they plan to channel their newfound control to create a positive life for themselves:

The world has good and bad, and this event has shown me a very bad side of the world, but I would rather focus on the good that happens, not ignore the bad but not dwell in it. This made my faith grow stronger.

## 4 | Discussion

The goal of the current study was to explore positive and negative life changes in exonerees, with a focus on worldview changes through the lens of Shattered Assumptions Theory. We aimed to understand worldview changes that occur following wrongful conviction in order to inform therapy modalities (e.g., CPT) that explicitly target cognitive changes related to worldview. This study was the first to explore the application of the Shattered Assumptions Theory to people who were wrongfully convicted and subsequently exonerated. Exonerees in the current study reported both positive and negative worldview changes, many of which exemplify the assumptions proposed in the Shattered Assumptions Theory (i.e., self is competent, people are benevolent, and the world is just and fair). This qualitative sample of 58 exonerees is the largest to date and therefore provides an exploratory foundation for future research exploring the clinical implications of worldview changes in exonerees. In addition to exploring worldview changes, life changes as a result of wrongful conviction were also explored, as it is essential to consider psychosocial context to provide effective clinical treatment and patient conceptualization. To provide context for worldview changes, we will first review the negative and positive life changes reported by participants. We will then discuss the specific worldview changes reported by participants, with a focus on how these worldview changes can be targeted through clinical interventions.

## 5 | Life Changes

### 5.1 | Negative Changes

When asked a neutral question about life changes as a result of their wrongful conviction, 78.5% of participants described negative changes. Almost half of participants described loss of relationships and social challenges as a result of their wrongful conviction, including difficulty forming close relationships, challenges trusting others, the end of friendships and romantic relationships, loss of family, and estrangement from children. Several exonerees described how their loss of relationships and social challenges were the most devastating impact of their wrongful conviction. Relatedly, many exonerees described how their wrongful conviction had a dire impact on their mental health, including posttraumatic stress, anxiety, depression, grief, lack of life satisfaction, difficulty sleeping, difficulty experiencing positive emotions, and emotional numbness. Many exonerees described how they have experienced negative changes to their worldview, including decreased faith in humanity and the legal system. Several exonerees described how they continue to struggle with the social and mental health impacts of their wrongful conviction decades after their release from incarceration, highlighting the need for long-term mental health services and social support for exonerees upon re-entry. As of 2022, only 12 states have statutes that provide mental health counseling for exonerees (Innocence Project 2023); results from this study highlight the need for statutory reform to ensure that exonerees across the United States have access to needed mental health treatment.

It is also important to note that approximately one-third of participants described experiencing structural barriers upon re-entry, ranging from challenges finding housing, barriers to employment, loss of career, financial problems, and a general lack of resources. Several exonerees described how the high profile nature of their cases led to the stigma and disgraced their reputation, further exacerbating the structural barriers experienced upon re-entry. These structural barriers experienced by exonerees highlight the need for programs and resources upon re-entry to help exonerees navigate challenges with obtaining housing, employment, and other social needs. Widespread integration of social workers into innocence organizations could be a helpful step towards addressing structural barriers faced by exonerees. One example is the Social Work Department at the Innocence Project, in which a team of social workers help exonerees with a variety of re-entry needs, ranging from obtaining forms of identification to securing housing, employment, and healthcare (Nguyen 2023).

### 5.2 | Positive Changes

When answering the neutral question about life changes as a result of their wrongful conviction, a striking 42.8% of participants described positive changes as a result of their wrongful conviction. It is important to note that posttraumatic growth does not indicate a lack of posttraumatic stress; in fact, many researchers theorize that posttraumatic growth occurs through the process of healing from posttraumatic stress, and thus growth cannot occur without stress (Tedeschi and Calhoun 2004).

In the current study, exonerees described all five domains of posttraumatic growth as defined by Tedeshi and Calhoun (1996), including improved relationships with others, identification of new possibilities for one’s life, increased perceptions of personal strength, enhanced appreciation of life, and spiritual growth. The most commonly reported positive changes included increased positive psychological changes, including increased appreciation for life, positive emotions, and a greater sense of meaning and purpose in life. Many exonerees described a newfound appreciation for relationships and closeness with others upon their release. This positive change is critically important, as social support is a strong predictor of positive mental health outcomes during the re-entry period (Fahmy and Testa 2025). Several exonerees described how their wrongful conviction led to a new career path, with several participants entering new careers in the legal field with the hopes of reforming the legal system to prevent future wrongful convictions.

Given that many participants described experiences of post-traumatic growth as a result of their wrongful conviction, it is important to consider how future research can examine factors that lead to positive outcomes in exonerees (rather than only focusing on factors that increase risk of negative outcomes). Many exonerees described a newfound motivation to help others and change the legal system, which highlights the potential for exonerees to serve in peer support roles to help other exonerees who are struggling upon re-entry. A significant body of research has revealed the benefits of peer support programs for people with histories of incarceration, including benefits to the peer support specialist and the client whom they are helping (Boles et al. 2022; Barrenger et al. 2020). Although it has been posited that exoneree peer support programs may help with reintegration upon reentry (Cummings et al. 2021), no research to date has examined the efficacy of such programs in exonerees. Future research should examine the efficacy of peer support programs for exonerees, as these programs could allow exonerees to share their experiences of healing and posttraumatic growth.

## 6 | Worldview Changes

When asked about changes to their worldview as a result of their wrongful conviction, participants described both negative and positive worldview changes. In terms of negative changes to worldview, the most commonly reported themes were loss of faith in the legal system, in people, and in authority figures. Other themes included loss of religious faith and several lessons learned as a result of their wrongful conviction (that problems exist in the legal system; that people are motivated by self interest; and that life is not fair and the world is not safe). Negative changes to worldview reported by exonerees tended to relate to the three central worldviews of the Shattered Assumptions Theory. Within the assumptive world of the Shattered Assumptions Theory, people are seen as benevolent and the world as just and fair. Common themes in worldview changes for this sample of exonerees revealed that these assumptive worldviews were significantly disrupted. Rather than believing in people as good, exonerees in this sample lost trust in people generally and felt betrayal by authority. For instance, one participant reported being “less trusting of people and the criminal justice system.” Another participant expressed that “there is no higher power and there are people in power and they are mostly only looking out for themselves.” Both quotes demonstrate a shattering of the assumption that people are benevolent. Most participants revealed distrust in the criminal legal system as a whole, suggesting additionally that their wrongful conviction may have challenged the assumption that the world is just and fair.

In terms of positive changes to worldview, themes included strengthened religious faith, learning that people are willing to help, and learning to control one’s attitude and focus on the positive. Consistent with the Shattered Assumptions Theory, one of the themes, learning that people are willing to help, demonstrates positive assumptions about peoples’ benevolence. One participant stated that “there are [people in power] who are willing to help and assist you,” which demonstrates a belief in the benevolence of people and that the world is just and fair. Although some participants described how their wrongful conviction shattered their sense of religious faith, other participants described how their sense of faith has strengthened as a result of their wrongful conviction. For example, one participant stated, “I became spiritual and learned to trust God.” Finally, several participants describe a newfound sense of control of their attitude as a result of focusing on the positive, which relates to the belief that the self is competent. Overall, several participants reported positive changes in their assumptions and attitudes about people and the world, consistent with the assumptions proposed in the Shattered Assumptions Theory.

The current results have important clinical implications for clinicians who provide psychotherapy to exonerees. Research has established the role of negative posttraumatic cognitive changes, such as negative changes to one’s worldview, in the development of PTSD and other psychopathology (Dunmore et al. 2001; Ehlers and Clark 2000; Foa et al. 1999; LoSavio et al. 2017; Schuler and Boals 2016; Gonzalo et al. 2012; Horwitz et al. 2018). Thus, many forms of cognitive therapy for PTSD, such as cognitive processing therapy (CPT), focus on identifying and modifying negative changes to one’s worldview as a result of their traumatic experiences (Resick et al. 2017). Research has supported the efficacy of CPT and other cognitive therapies in addressing PTSD symptoms related to a variety of trauma types (Roberge et al. 2022; Lee and Bowles 2020). However, research has revealed that the efficacy of PTSD treatments varies depending on the trauma type (e.g., interpersonal trauma, combat trauma, experiencing a natural disaster, etc.), and thus it is important to consider ways that cognitive therapies can be adapted to consider the trauma types that are commonly experienced by exonerees (Lee and Bowles 2020).

Results from the current study provide preliminary evidence for commonly reported worldview changes in exonerees to target in cognitive therapy for PTSD in exonerees. Specifically, exonerees frequently reported loss of faith and trust in people more generally and in the legal system and the actors within the legal system. Exonerees also frequently reported several global changes to worldview, such as the newfound belief that the world is not safe and that life is not fair. The variety of worldview changes experienced by exonerees demonstrate how being wrongfully convicted is a unique type of trauma, as it often involves trauma on the interpersonal (i.e., actors within the legal system who caused or condoned the wrongful conviction) and structural (i.e., the legal system) levels, as well as chronic trauma exposure over a lengthy period of time. Little research has examined evidence-based treatments for PTSD in people coming home from incarceration more generally (Pettus 2023), and no research has established evidence-based trauma treatments for exonerees. This study is the first to map on specific cognitive changes to worldview to Shattered Assumptions Theory in order to inform PTSD treatments that target posttraumatic cognitive changes. Future research should explore ways of adapting existing cognitive therapies for PTSD to ensure that they properly address exonerees’ unique experiences and worldview changes.

This study is not without limitations. First, the study utilizes open-ended survey responses, which does not provide the same level of in-depth data as a qualitative interview. Although the depth of data is limited, open-ended survey questions can be a useful approach to gaining a preliminary understanding of sensitive or personal topics, as participants may share information more openly (Sheldon 2017; Albudaiwi 2017; Fisher 1993). Building on the results from this preliminary study, future studies should conduct in-depth qualitative interviews of exonerees to further explore changes in worldviews as a result of their wrongful convictions. Second, the study participants are not racially representative of exonerees as a whole, as only 19.0% of participants identified as Black while 53% of the exoneree population is Black (Gross et al. 2022). Future research should examine the unique experiences of exonerees of color, including the impact of experiences of structural racism on their mental health and reentry experiences. Third, the sample may not be representative of exonerees as a whole given that participants were recruited through an online listserv and data was collected through an online survey. Not all exonerees are members of the listserv that was used to recruit participants, and participants’ ability to complete the survey may have been impacted by their access to and comfort with technology. Future studies can utilize more comprehensive approaches to participant recruitment beyond online listservs. For example, in future studies, research staff can assist participants in completing the survey to ensure that technology issues do not prevent participants from completing the survey. Fourth, while efforts were made to use simple language, it is possible that reading comprehension issues may have prevented some potential participants from completing the survey. Finally, the survey is based on retrospective, self-reported reflections from a single time point, and thus the results may be impacted by recall bias or social desirability bias.

## 7 | Conclusion and Strengths

The study also has several strengths, with one of them being the sizable number of participants in a hard-to-reach population (*n* = 58). While the open-ended survey method is noted as a limitation, it may have allowed participants to disclose more than they would have in a live interview due to the sensitive nature of both their experiences and the question. While the participants in this study were not racially representative of exonerees as a whole, other legal variables (e.g., length of incarceration and number of appeals) had significant variability, and thus the sample is generalizable to a variety of legal experiences. Even with our limitations, this study addresses a critical gap in the literature. Exonerees have a significant need for mental health services given the high rates of PTSD and other mental health disorders, but little research has focused on adapting existing evidence-based treatments to meet the unique needs of exonerees. The results and integration of theory in this study provide important inlets for clinicians and researchers to further understand the unique experiences of exonerees in a way that can meaningfully inform clinical treatment and future research.

## Figures and Tables

**TABLE 1 |:** Demographic characteristics of participants.

Demographic characteristics	*n* (%)
Gender	
Male	44 (75.9)
Female	14 (24.1)
Highest level of education	
High school	24 (41.4)
College	22 (37.9)
Advanced degree	12 (20.7)
Race	
American Indian or Alaska Native	1 (1.7)
Asian or Pacific Islander	1 (1.7)
Black	11 (19.0)
Hispanic	6 (10.3)
White	38 (65.5)
Multiracial	1 (1.7)
Marital Status	
Single—Not dating	18 (31.0)
In a relationship—Not married	20 (34.5)
Married	20 (34.5)
Current employment Status	
Employed	30 (51.7)
Not employed	27 (46.6)
Religious/Spiritual	
Religious/Spiritual	40 (69.0)
Not religious/Spiritual	18 (31.0)

**TABLE 2 |:** Legal variables.

Legal variables	*n* (%)
Number of trials	
One	45 (77.6)
Two	11 (19.0)
Three	1 (1.7)
Four or more	1 (1.7)
Type of attorney	
Private attorney	34 (58.6)
Public defender	22 (37.9)
Represented self *Pro Se*	1 (1.7)
Released on bond	
Yes—Released on bond	24 (41.4)
No—Not released on bond	34 (58.6)
Number of appeals	
One	12 (20.7)
Two	9 (15.5)
Three	6 (10.3)
Four	3 (5.2)
Five or more	27 (46.6)
Exonerated In state or federal court	
State court	50 (86.2)
Federal court	7 (12.1)

**TABLE 3 |:** Frequency of themes and subthemes.

Themes and subthemes	*n* (%)
Question 1	
Positive changes	
Positive psychological changes	11(19.6%)
Increased appreciation of life	8 (14.2%)
Positive emotions	3 (5.4%)
Greater sense of meaning	2 (3.6%)
Improved relationships	7 (12.5%)
Increased appreciation of family	4 (7.1%)
Increased closeness with others	3 (5.4%)
Increased spirituality	8 (14.2%)
Positive career changes	5 (8.9%)
Became a better person	2 (3.6%)
Negative changes	
Loss of relationships	27 (48.2%)
Structural barriers	18 (32.1%)
Grief/Loss	15 (26.7%)
Loss of Parenting	12 (21.4%)
Negative emotions & psychological changes	10 (17.9%)
Financial problems	5 (8.9%)
Negative changes to worldview	5 (8.9%)
Disgraced reputation	5 (8.9%)
Health problems	3 (5.4%)
Arrested development/Loss of formative years	2 (3.6%)
Neutral changes	
Every level changes	12 (21.4%)
Question 2	
Mental health consequences	31 (55.4%)
Social problems	22 (39.3%)
Structural barriers	20 (35.7%)
None	8 (14.3%)
Stigma	6 (10.7%)
Question 3	
Mental growth	16 (28.6%)
Relational growth	14 (25.0%)
Purpose	12 (21.4%)
New skills	12 (21.4%)
Spiritual growth	11 (19.6%)
None	10 (17.8%)
Question 4	
Negative changes to worldview:	
Loss of faith in legal system	11 (21.2%)
Loss of faith/Trust in people	8 (15.4%)
Loss of faith/Trust in authority Figures	7 (13.5%)
Loss of religious faith/Challenges to religious beliefs	5 (9.6%)
Learned about wrongful convictions/Problems with legal system	4 (7.7%)
Learned that people are motivated by self-interest	3 (5.8%)
Learned that life is not fair & world is not safe	3 (5.8%)
Positive changes to worldview:	
Strengthened religious faith	5 (9.6%)
Learned that people are willing to help	4 (7.7%)
Learned to control attitude/Focus on positive	2 (3.8%)

## Data Availability

The data that support the findings of this study are available from the corresponding author upon reasonable request.
